# Loneliness and social conformity: A predictive processing perspective

**DOI:** 10.1111/nyas.15324

**Published:** 2025-04-02

**Authors:** Naem Haihambo, Dayo‐Marie Layiwola, Helen Blank, René Hurlemann, Dirk Scheele

**Affiliations:** ^1^ Department of Social Neuroscience, Faculty of Medicine Ruhr University Bochum Bochum Germany; ^2^ Research Center One Health Ruhr of the University Alliance Ruhr Ruhr University Bochum Bochum Germany; ^3^ Predictive Cognition, Faculty of Psychology Ruhr University Bochum Bochum Germany; ^4^ Department of Psychiatry, School of Medicine & Health Sciences University of Oldenburg Oldenburg Germany; ^5^ Research Center Neurosensory Science University of Oldenburg Oldenburg Germany

**Keywords:** chronicity, internal model, loneliness, oxytocin, social cognition, social conformity

## Abstract

For social creatures like humans, loneliness—which is characterized by a perceived lack of meaningful social relationships—can result in detrimental health outcomes, especially when experienced over an extended period of time. One potential way to pursue rewarding social connections could be social conformity, the tendency to align one's behavior and opinions to those of others. In this perspective article, we give a broad overview of common and distinct neural mechanisms underlying loneliness and social conformity, and the involvement of the oxytocinergic system therein. Additionally, we consider how loneliness can be understood within a predictive processing framework. Specifically, negative expectations could be related to altered representations of the self and others in the medial prefrontal cortex, whereas diminished bottom‐up signals from the insula may contribute to reduced precision in the perception of the social environment. This negatively skewed internal model may perpetuate loneliness and lead to chronicity over time. While acute isolation and loneliness could drive people toward reconnection and increased social conformity, chronic loneliness may lead to distrust and avoidance, eventually resulting in nonconformity. We suggest different mediating mechanisms and moderating factors that warrant further investigation in future research.

## Graphical abstract

Loneliness arises from a perceived lack of meaningful social connections, shaping both perception and behavior. While acute loneliness may promote social conformity as a means of reconnection, chronic loneliness can foster distrust, withdrawal, and non‐conformity. From a predictive processing perspective, altered self–other representations in the medial prefrontal cortex reduces precision in social perception, and dysregulation of the oxytocinergic system may reinforce loneliness, which may contribute to its chronicity over time.

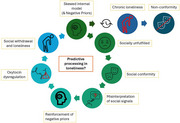

## INTRODUCTION

Humans are social creatures driven by an inherent need for connection and belonging. Loneliness, often experienced as a yearning for meaningful relationships, is a poignant manifestation of this innate drive for interpersonal connection.^1^ When we lack a sense of belonging or do not find meaningful rewards in social interactions, loneliness compels us to seek out social connections as a means of alleviating this distress. Chronic loneliness is associated with adverse health outcomes, including cognitive decline and premature mortality.^2^, ^3^

In the pursuit of connection, individuals have been found to engage in behaviors aimed at gaining social acceptance and belonging.^4^ One such behavior could be social conformity, defined as the adjustment of one's beliefs, attitudes, or behaviors to align with those of a group.^5^ Social conformity fosters group cohesion and acceptance. By aligning with group norms, individuals may gain a sense of security and inclusion, which can alleviate feelings of isolation.^6^ While other variables, such as personality traits, also influence loneliness,^7^ social conformity offers unique insights into behavioral adaptations to social feedback.

This adjustment toward social norms shares conceptual similarities with reinforcement learning. Both processes rely on neural mechanisms in the midbrain and sociocognitive brain regions, including the prefrontal and anterior cingulate cortex.^8^, ^9^ These systems process mismatches between the brain's expectations and actual outcomes (i.e., prediction matches and errors). Although debated,^10^ recent theoretical frameworks conceptualize loneliness as a dysfunction in regulatory mechanisms including the error‐detection system, which monitors and predicts alignment between self (internal) and other (external).^9^, ^11^

This perspective article seeks to identify potential converging neural underpinnings of loneliness and social conformity and understand how loneliness can be explored within the predictive processing framework, which views the brain as continuously monitoring, generating, and updating predictions about the environment based on prior and ongoing experiences. While previous theories, such as cognitive bias models, emphasized distorted and negative perceptions in loneliness,^12^ we propose that predictive processing expands these static biases and offers a more dynamic and context‐sensitive approach across multiple levels of cognition and behavior. This framework is particularly suited for exploring how loneliness and social conformity change over time.

Describing neuroimaging findings adds significant value by providing neurobiological evidence of the brain regions and mechanisms involved to potentially validate theoretical models and develop targeted interventions. Furthermore, we discuss potential differences in neural activation and processing due to the stage of loneliness (acute vs. chronic). Predictive processing may unify cognitive and emotional processes at behavioral and neural levels, offering a holistic understanding of dynamic responses to social environments, which is more context sensitive. This approach also offers testable hypotheses discussed later in this perspective.

We will first introduce the concept of predictive processing, followed by broad neurocognitive mechanisms of loneliness and social conformity from human research. Loneliness is a psychological state that involves complex interactions between brain function and social behavior.^13^ Neural systems that process social signals and regulate emotions—such as the anterior cingulate cortex, insula, prefrontal cortex, and amygdala—shape the experience of loneliness.^12^, ^13^, ^14^ Dysregulation in these regions has been linked to heightened sensitivity to social rejection and impaired reward processing, which are central to the development and maintenance of loneliness.^14^

Various hormonal, molecular, and neuropeptide systems are involved in these complex constructs. Among these, the hypothalamic peptide, oxytocin, is implicated in both loneliness and social conformity. For example, research has linked loneliness to dysregulated oxytocin signaling,^15^ which may contribute to negative emotional and cognitive biases like heightened vigilance to social threats and reduced perception of social rewards.^12^, ^15^, ^16^

We conclude by proposing a model that integrates our hypotheses and mechanisms mediating the association between loneliness and social conformity within the framework of predictive processing.

## PREDICTIVE PROCESSING, INTERNAL MODELS, AND THE BRAIN

The brain has been hypothesized to function as an adaptive prediction machine that continuously anticipates sensory input based on prior experience.^17^, ^18^, ^19^ Our everyday world is dynamic and provides noisy and ambiguous sensory inputs. To navigate this uncertainty, according to frameworks of predictive processing, the brain constructs predictive models to anticipate future events and infer their causes.^19^, ^20^ This process involves combining prior knowledge with sensory data to create our social and perceptual experiences, allowing the brain to adapt flexibly to changing conditions.

Within the hierarchical framework of predictive processing, higher‐level representational units produce top‐down predictions about expected sensory information.^21^ These predictions are matched against sensory input by lower‐level error units, which calculate the prediction error. These errors may be essential for adjusting prior expectations about incoming sensory information, thus enhancing prediction accuracy. These generative models may function as cognitive templates, simulating and predicting the outcomes of our actions and perception.^22^

### Predictive processing and social cognition

While predictive processing has traditionally been studied for sensory perception, recent research highlights its critical role in social information.^23^, ^24^ The brain not only predicts physical sensory information but also makes inferences about social cues, intentions, and outcomes.^25^ For example, individuals construct internal models of the self shaped by their experiences and relationships.^26^ These internal models enable anticipation of how one will be perceived, consequences of social behaviors, and adaptation. Such models are continuously constructed and updated, integrating feedback to shape expectations about group dynamics, social rewards, and interactions.

When incoming information matches model predictions, this confirmation consolidates the model. In contrast, perceptual deviations and mismatch result in a prediction error.^27^ It has been suggested that the goal of the predictive process is to minimize prediction errors. This could be implemented in various ways. First, the internal model could be updated based on the prediction error.^28^ Second, according to accounts of active inference, the motor system could adjust the behavior for better alignment of the incoming stimuli with our predictions and internal model.^29^ Finally, deviating sensory signals may be discarded and biased by prior expectations to match the internal model.^30^ The degree to which expectations shape the resulting posterior percept depends on the strength or precision of both perceptual priors and sensory input.^31^ The precision of our predictions might be based on factors like the consistency of our environment or the reliability of certain cues preceding specific events. Less precise priors have a reduced effect on the representation and perception of sensory information, while more precise priors exert a greater influence.^18^

A predictive processing account of loneliness has not been formalized, though it has been proposed that loneliness may arise from a persistent inability to reconcile expectations with reality.^30^ and consistent negative social expectations.^32^ Building on this idea, dysregulation in social reward processing and cognitive biases could contribute to maladaptive predictions and distorted internal models. These disruptions may create a feedback loop where negative expectations shape behaviors that reinforce feelings of isolation, particularly when these patterns persist over time.

### Neural substrates of predictive processing

Neurally, the default mode network (DMN), which strongly overlaps with areas responsible for social cognition,^33^, ^34^ seems to contribute to predictive processes by making probabilistic estimations of future events based on prior and imagined events.^35^, ^36^, ^37^ Although resting state and cognitive functions do not specifically overlap, the DMN seems to be activated at wakeful rest when we mind‐wander, think about the past, and daydream about the future,^35^ highlighting its role in mentalizing and social cognition. The DMN comprises interconnected brain regions, including the temporoparietal junction, precuneus, areas of the cingulate cortex, and more. However, for brevity, we focus on the largest areas of the DMN, including both ventral and dorsal areas of the medial prefrontal cortex (mPFC).^34^, ^38^ Previous studies have implicated the ventral mPFC (vmPFC) in social prediction errors and social appropriateness,^39^ while the dorsal mPFC (dmPFC) has been associated with emotion expression, social decision‐making, and imagining future outcomes.^40^ As an integrated hub in the DMN, the mPFC is said to generally engage in self‐referential processing, introspection, and social cognition.^41^, ^42^ Predictive processing theories propose that the ventral and dorsal mPFC generates top‐down predictions based on internal models, allowing us to anticipate and adapt to environmental changes even during moments of rest.^33^, ^35^, ^43^, ^44^

The posterior cerebellum, which is functionally connected with most areas in the social cognition network,^45^ has been associated with making sociocognitive predictions, especially when sequences of events and timing are involved.^25^, ^46^, ^47^, ^48^ While inconsistent findings exist regarding the cerebellum, some meta‐analyses report activation in this region,^49^ while others do not find cerebellar involvement during predictive processing.^50^

The bilateral insula is consistently implicated in task‐based predictive processing, often in conjunction with the DMN.^44^, ^49^, ^50^ Interestingly, the insula has been found to play a role in interoceptive awareness (i.e., our sense of internal body states), which it integrates with external input and social cues.^51^ This integration is critical for error detection, allowing the brain to reconcile mismatches between internal states and external feedback.^31^, ^52^ The contribution of these brain regions to predictive processing is summarized in Table 1.

**TABLE 1:** Summary of selected brain regions, their relevant functions, and their hypothesized contributions to the reinforcement and adjustment of internal models in social cognition and loneliness.

Brain region/network	Relevant function	Hypothesized contribution to the internal model
**Amygdala**	Emotional processing, fear response	The amygdala processes emotions, particularly fear, and impacts behavior by regulating anxiety, aggression, stress responses, and social cognition. It adjusts internal models based on emotional experiences and responses.
**Default mode network (DMN)**	Social cognition, self‐referential thought	The DMN is involved in understanding others’ mental states and self‐other distinctions. It adjusts internal models based on new social information and self‐reflection.
**Insula**	Emotional processing, interoception	The insula processes emotions and bodily states, contributing to internal model adjustments by integrating interoceptive and emotional signals.
**Medial prefrontal cortex** **(mPFC)**	Social cognition, decision‐making	The mPFC plays a key role in social understanding and empathy. It reinforces and adjusts internal models through social interactions and decision‐making processes.
**Posterior cerebellum**	Social cognition, timing	The cerebellum acquires and stores internal models about the timing of social and nonsocial information.

*Note*: This framework integrates theoretical perspectives and highlights potential pathways for future empirical investigation.

The neuropeptide oxytocin has been extensively studied for its role in modulating attachment, trust, and social salience processing.^53^ While the exact effects of oxytocin on predictive processing remain unclear, it appears that oxytocin modulates sensitivity to environmental changes and social dynamics.^54^ Oxytocin potentially influences predictive processing by directing attention to important social information and increasing the precision of internal models. We speculate that individuals with impaired oxytocin reactivity, such as lonely individuals,^30^, ^55^, ^56^ may struggle with adapting to dynamic environments, reducing the likelihood of internal model updates. In the next section, we will explore the neural substrates of loneliness and how they relate to predictive processing.

## LONELINESS AND ASSOCIATED NEURAL SUBSTRATES

**FIGURE 1:**
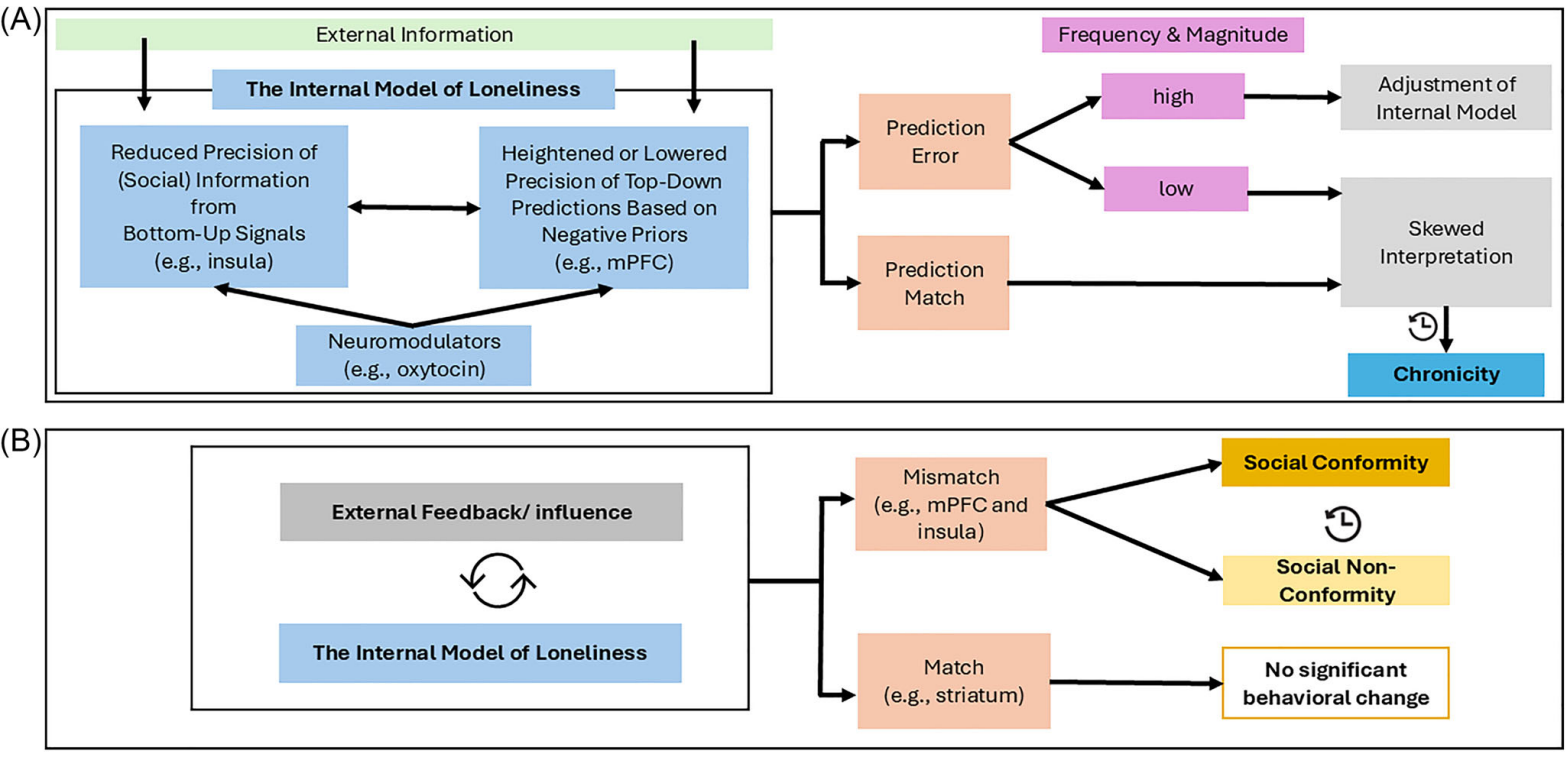
(A) Depiction of the internal model of loneliness and its interaction with external information and feedback. External information influences the internal model, characterized by reduced precision of social information from bottom‐up signals (e.g., insula) and varied precision of top‐down predictions based on negative priors (e.g., mPFC). Neuromodulators like oxytocin play a role in modulating these processes. The internal model generates a prediction error or match. Frequent and significant prediction errors lead to either adjustments of the internal model or a skewed interpretation, contributing to the chronicity of loneliness. (B) The bidirectional interaction between external feedback and the internal model of loneliness. External feedback can either match or mismatch the internal model's predictions. A match results in no significant behavioral change, while a mismatch may be linked to conformity or nonconformity depending on the gradient from lonely to chronically lonely (this is further elaborated in Figure 2). Moderators such as attachment style, sex, and type of influence (normative vs. informational) play a role in this process.

From a predictive processing view, loneliness has been described as a state where the brain's expectations about social connections do not match reality, resulting in persistent prediction errors.^30^ In this light, loneliness might be the result of the brain continuously anticipating a certain level or quality of social interaction that is not met. While predictive processing theory suggests that initial mismatches between expected and actual social experiences should lead to adjustments in the brain's internal model to minimize prediction errors, this process may become disrupted in loneliness. In lonely individuals, a rigid or pessimistic internal model may resist adaptation, leading the brain to skew interpretations of social interactions in ways that confirm existing expectations of rejection or isolation, which maintain internal models without updating them (see Figure 1A).

### Loneliness and introspective processing

Loneliness is also conceptualized as an allostatic overload, where the stress of lacking social connections disrupts the body's ability to maintain stability through change. This disruption appears to lead to incorrect predictive processing of internal bodily sensations that help us understand our own physiological state.^57^ Accumulating evidence suggests altered activity and connectivity of the insular cortex in loneliness. For example, lonely individuals reporting decreased interpersonal trust exhibit reduced insula activation during trust decisions.^56^ While this study did not include feedback to directly investigate predictive processes, the reported insula hypoactivation could reflect diminished production of, or sensitivity to, bottom‐up signals. As the insula integrates internal bodily states with external social cues,^44^, ^58^ diminished sensitivity may impair the accurate interpretation of social signals or appropriate emotional responses to interactions. Reduced functional connectivity between the insula and occipitoparietal regions was correlated with diminished affective responsiveness to positive social interactions,^56^ which might reflect difficulties in recognizing introspective signals. Furthermore, insula responses to emotional stimuli mediated the link between alexithymia and loneliness,^59^ suggesting that altered interoceptive processing contributes to perceived social isolation.

Higher loneliness was associated with more negative expectations of partners’ trustworthiness and a stronger weighting of negative information about initially dishonest partners.^60^ It is, therefore, possible that when a prediction match occurs between the expectation that others cannot be trusted and the observation of dishonest behavior, a negatively skewed internal model might be strengthened, reinforcing a stronger negatively weighted bias.

### DMN in loneliness

Research indicates that loneliness is associated with differences in the DMN. For example, loneliness‐related changes are evident in the connectivity between the insula and DMN.^61^ Additionally, increased cerebellar volume, activation, and connectivity to areas of the DMN positively correlated with loneliness susceptibility during isolation.^62^ Interestingly, while watching video clips, lonely individuals exhibited responses in areas of the DMN that differed from those of their peers.^63^ In line with this, increased loneliness was associated with reduced neural similarity of self and other representations in the dmPFC.^64^ Furthermore, neural representations in the dorsal and ventral mPFC and precuneus/posterior cingulate cortex distinguishing real and fictional others were also blurred in lonely individuals.^65^ Thus, in addition to disruption in insular‐mediated, bottom‐up interoceptive signals, the internal model of loneliness may also include biased mPFC‐driven predictions.

Loneliness and social anxiety have been shown to be positively associated and are reciprocally associated over time.^66^ Individuals with high social anxiety showed reduced subjective value of social engagement and increased amygdala responses in a monetary gamble task with social feedback.^67^ In contrast, Bayesian analyses provided evidence for the absence of meaningful behavioral or neural differences between individuals with high and low loneliness scores in the same task.^68^ Unlike social anxiety, which is often associated with heightened amygdala reactivity, loneliness may not consistently involve hyperactivity in threat‐detection areas.^69^ Additionally, loneliness was not significantly associated with amygdala activation in response to social compared to nonsocial scenes.^70^

A more recent study found that lonely men, but not women, demonstrated reduced amygdala habituation to repeated fearful faces and amygdala hyperreactivity during fear conditioning.^71^ Further research suggests possible sex‐specific effects of loneliness. For instance, men demonstrated a stronger correlation between smaller amygdala volumes and higher loneliness scores.^72^ as well as alterations in the DMN.^61^

### Reward processing and loneliness

A plethora of studies provide evidence for the involvement of reward‐associated brain regions in social bonding and attachment. The reward system—which includes regions like the striatum, ventral tegmental area, nucleus accumbens, hypothalamus, and basal forebrain—is essential for processing rewards and reinforcing behaviors that promote social bonding, attachment, and survival.^73^, ^74^, ^75^, ^76^, ^77^ While there is an overlap in neural representations of primary and secondary rewards, there is also evidence for reward type–dependent activation.^78^ Oxytocin has been shown to enhance a positive partner bias, accompanied by increased neural activation in the nucleus accumbens,^73^ potentially pointing to a synergistic role of oxytocin and areas of the reward system in bonding and attachment.

Loneliness has been linked to reduced affective and oxytocinergic responses to positive social interactions,^56^ but dysfunctions of reward circuits were not consistently observed. Lonely individuals displayed weaker striatal activation when viewing social stimuli compared to objects with pleasant depictions.^58^ However, other studies failed to detect significant alterations in reward‐associated brain activity related to loneliness,^70^ or observed altered responses only for pictures depicting close others.^80^

### Oxytocin and loneliness

Oxytocinergic response to positive social interactions is significantly reduced in lonely individuals.^56^ Movement synchronization in dyads induces the release of oxytocin.^80^ and changes in oxytocin levels predicted reduced loneliness in cases where older adults experienced high levels of closeness and synchrony during a mirror game.^55^ However, the administration of intranasal oxytocin before a group psychotherapy session against loneliness did not significantly enhance the intervention effects on trait‐like loneliness.^82^ Of note, the same study found that oxytocin facilitated the decrease in state loneliness within therapy sessions and improved positive bonding between the group members.

### Acute and chronic loneliness

Detrimental effects of loneliness predominantly occur when it is experienced over an extended period of time. Therefore, a distinction between the effects of acute loneliness versus chronic loneliness must be made.^82^, ^83^ Acute social isolation is associated with lowered self‐reported energetic arousal and heightened fatigue,^84^ feelings that are not typically present in chronic loneliness. Acutely lonely individuals have been shown to prefer smaller interpersonal distances.^85^ In contrast, chronically lonely individuals have been found to prefer larger interpersonal distances, hinting at a withdrawal from social interaction rather than a desire for it.

Along these lines, despite high levels of social anxiety in lonely individuals, they did not show typical avoidance behavior in a social gambling task or demonstrate amygdala hyperreactivity,^68^ which is commonly observed in individuals with social anxiety. It is, however, conceivable that such patterns emerge after some time. Alternatively, contextual factors may moderate the effects of loneliness. For instance, inclusion motivation was heightened in lonely individuals only during fair but not exclusionary interactions.^86^

Taking chronicity into account could also help to explain the paradoxical finding that individuals with higher loneliness scores had less favorable trustworthiness expectations but trusted their partners to a greater extent in economic games.^87^ One potential explanation could be that this discrepancy between trust expectations and actions represents a conformity response. Lonely individuals might engage in trusting behaviors because they perceive such actions as socially appropriate or expected, even if these actions contradict their internal expectations. This dissonance between behavior and expectations could contribute to chronic disappointment if the outcome of trusting behavior is perceived as negative. If loneliness persists over longer periods of time, negative expectations about others may result in negative behavior. For example, lonely people often display insecure attachment styles, characterized by anxiety, fear of abandonment, and difficulty trusting others.^88^ Similarly, the magnitude and duration of loneliness could foster an increase in aggressive behavioral tendencies or a lack of inhibition of aggressiveness.^89^

## SOCIAL CONFORMITY

Social conformity refers to the tendency of individuals to adjust their thoughts, feelings, and behaviors to align with group norms, societal expectations, or cultural standards.^4^ Studies have demonstrated that adults may abandon their perceptual judgments when a group of confederates unanimously disagreed with them in a simple visual task.^5^ This phenomenon highlights the potential of social influence on personal judgments and behavior, which has been repeatedly confirmed across different studies with variable group size,^5^, ^90^ task difficulty and contextual relevance,^91^ personal motivation,^92^ and emotional state.^93^ Conformity is primarily driven by two types of influence: normative and informational. Normative influence involves conforming to group norms to gain social acceptance or avoid rejection, whereas informational influence occurs when individuals conform because they believe that others possess more accurate or reliable information;^4^, ^94^ both types of conformity have been shown to be associated with activation in brain regions, such as the mPFC.^95^ Furthermore, individuals may also conform to maintain a positive self‐image.^4^, ^96^, ^97^

Neuroimaging studies indicate that social conformity engages multiple neurocognitive processes, including brain networks implicated in conflict, reward processing, and mentalizing.^9^, ^98^, ^99^ Specifically, the anterior insula was consistently activated when people were processing the discrepancy between their own choices and those of a group,^100^, ^101^ as well as when complying with group norms.^102^ Additionally, brain reward regions such as the striatum are recruited when individuals adjust their behavior in line with group norms,^8^ potentially reflecting the rewarding experience of feeling more accepted by the group. Finally, when people observe the violation of group norms, areas involved in mentalizing such as the temporo‐parietal junction, mPFC, and cerebellum are recruited.^103^ Similarly, activation in the dmPFC increased when people tracked discrepancies between personal preferences and social ideals.^104^

### Oxytocin in conformity

Accumulating evidence indicates that oxytocin plays a role in shaping social conformity, but specific effects differ between tasks and samples. For instance, oxytocin stimulated in‐group conformity, highlighting its influence on favoring group cohesion over individual dissent.^85^ Correspondingly, individuals with high levels of xenophobia significantly increased refugee‐directed donations if peer‐derived altruistic norms were paired with oxytocin.^105^ Furthermore, oxytocin enhanced implicit social conformity to both in‐group and out‐group opinions, suggesting a broad impact beyond mere in‐group favoritism.^87^ Interestingly, variation in the oxytocin receptor gene was linked to mPFC activation in the face of social misalignment.^106^ These results suggest that oxytocin may promote conformity by dampening the conflict‐related signal in the mPFC.

However, in the same way that the anxiolytic and prosocial effects of oxytocin differ between contexts and individuals,^107^ the impact of oxytocin on conformity is not unequivocal. In a decision‐making task, oxytocin had no significant overall effect, but participants with a high need for structure and high approach sensitivity violated group norms more often under oxytocin than placebo.^108^ In a coin‐tossing task, oxytocin significantly increased conformity, but only in a competitive environment.^109^ Furthermore, oxytocin enhanced immediate compliance with social norms but reduced the internalization of these influences into long‐term beliefs,^110^ suggesting a dissociation between overt behavior and internal belief changes under social pressure because of intranasal oxytocin.

### Do the lonely conform?

Combining the insights on loneliness and social conformity reveals significant overlaps. Both constructs engage similar neural substrates, such as the anterior insula, mPFC, and striatal structures, which are involved in predictive error and reward processing.^8^, ^11^, ^56^, ^59^, ^61^, ^111^ Additionally, both are influenced by the oxytocinergic system, despite some contradictory findings.^106^, ^112^, ^113^

Despite these commonalities, research directly linking loneliness and social conformity is scarce and presents mixed results. For instance, lonely individuals were less likely to comply with COVID‐19 preventive measures.^114^ Contrarily, another study reported a positive association between emotional loneliness and compliance, with social loneliness showing no significant link to preventive behavior.^115^ Furthermore, lonely individuals tended to prefer minority‐endorsed products in private settings but shift to majority‐endorsed products in public contexts.^116^ Similarly, lonely males demonstrated lower confidence and a reluctance to express their opinions publicly, while lonely females tended to show increased conformity and susceptibility to social influence,^117^ potentially demonstrating sex‐specific effects. Additionally, loneliness significantly mediated the likelihood of conformity to traditional norms among males with high depressive symptoms.^118^ Collectively, this research indicates that both individual and contextual factors shape how loneliness influences the need for belonging through conformity.

## PROPOSED MODEL OF PREDICTIVE PROCESSING AND SOCIAL CONFORMITY IN LONELINESS

Considering all this information, we propose a model (Figure 1) in which acute isolation and transient loneliness may lead to social conformity, particularly as a short‐term adaptive response to fulfill social connection needs. However, if loneliness becomes chronic, the inclination to conform may diminish as individuals develop avoidance behaviors or distrust in others.

We propose that loneliness may be explained as the result of a skewed internal predictive model. The skewed model may generate biased top‐down predictions rooted in negative priors, potentially generated in brain regions like the mPFC. Altered bottom‐up signals, particularly in areas such as the insula, may lead to diminished precision of social information. Neuromodulators like oxytocin may shape both top‐down and bottom‐up processes related to social perception.^119^ When social events are interpreted through these biased internal models, prediction errors can arise, necessitating adjustments to the model. However, if individuals who feel lonely are consistently exposed to a high magnitude and frequency of social cues that leave them feeling unfulfilled, or if their internal models are robust enough to automatically skew new information, they may experience ongoing prediction mismatches. Over time, this pattern of biased processing can contribute to the development of chronic loneliness. Thus, acute feelings of loneliness may result from unfulfilled social expectations (i.e., a mismatch between the brain's expectations about social connections and the reality),^30^ while chronic loneliness may be characterized by a biased internal model.

### The impact of chronicity on conformity

It is currently unclear when the transition from acute isolation to loneliness, and ultimately to chronic loneliness occurs. Loneliness measures, such as the UCLA Loneliness Scale.^120^ often do not include a time or chronicity measure. Nonetheless, we propose that this transition is a culmination of the intensity and frequency of unfulfilled social needs over time. We also propose that the association between loneliness and social conformity changes gradually, and transition points may differ between individuals. During acute isolation, individuals may experience reduced energetic arousal which is linked to social conformity.^121^ In a state of reduced energetic arousal, conformity may be increased as it may require less cognitive effort.^122^

Individuals with low trait interoceptive accuracy are more susceptible to social influence in dyadic interactions, although this effect diminishes when feedback is received.^123^ Given the evidence for reduced insula activity^56^ and heightened alexithymia in loneliness,^59^ we hypothesize that lonely individuals may exhibit reduced introspective awareness, impairing their ability to rely on internal signals, such as emotional or physiological states, when making decisions. This diminished reliance on internal cues may increase dependence on external social information, increasing susceptibility to social conformity as they seek validation or guidance from others to compensate for uncertainty.

Moreover, chronically lonely individuals exhibit reduced interpersonal trust and lower oxytocinergic reactivity to positive dyadic interactions.^56^ Research indicates that trust in others, including robots, can facilitate conformity; however, this tendency significantly diminishes when trust is undermined due to consistently incorrect information from these sources.^124^ Familiarity and trust also positively correlate with memory conformity.^125^ Distrust together with reduced oxytocin release may thus result in social nonconformity. Furthermore, given the reduced neural similarity of self and other representations in the mPFC,^64^ we suggest that chronic loneliness is associated with nonconformity because it is associated with an increased tendency to perceive others as out‐group members (Figure 1B).

We propose that, over time, the increasing characteristics of loneliness may lead to a decrease in conformity. This gradual decline can manifest as a tendency to avoid social interactions,^85^ ultimately diminishing inhibition of aggressive tendencies as loneliness becomes chronic. Thus, this chronic state may further reduce the likelihood of social conformity, favoring avoidance and nonconformity as individuals withdraw from social engagement.

### Task‐related moderaters for conformity in loneliness

**FIGURE 2:**
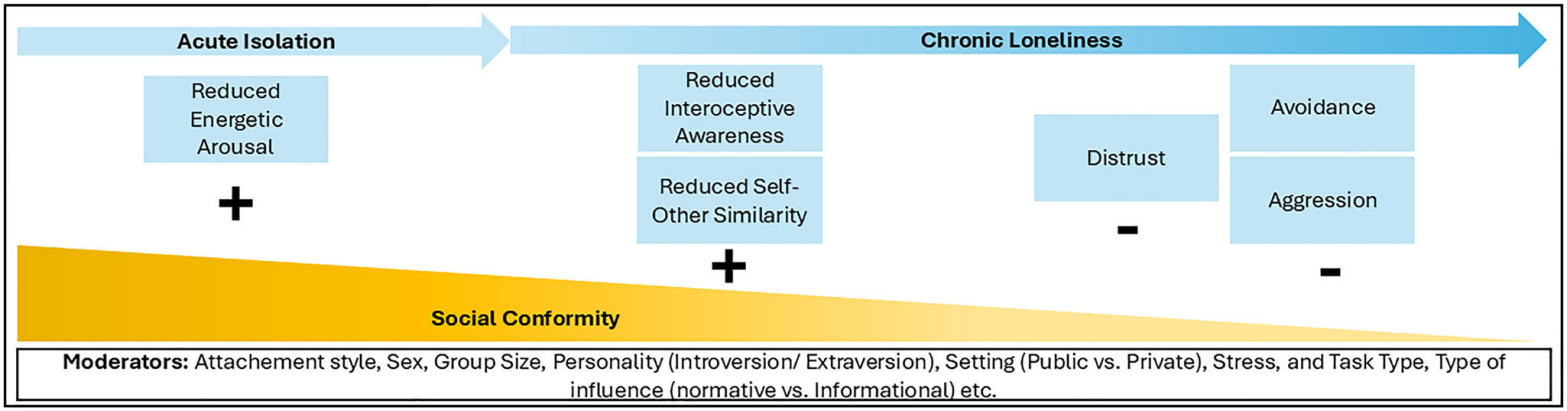
The probability of displaying social conformity behavior, ranging from acute isolation to loneliness to chronic loneliness. The color gradient from light to dark blue indicates increasing chronicity of loneliness over time, while a gradient from dark to light orange represents a decreasing probability of displaying social conformity. The nonexhaustive list of processes and factors enhancing the probability of displaying social conformity is marked with a plus (“+”), and social conformity–reducing processes and factors are marked with a minus (“−”). The nonexhaustive list of moderators refers to additional factors that may influence the display or probability of social conformity.

It is important to acknowledge that several potential moderators could impact both neural and behavioral conformity in lonely individuals (Figure 2). For example, the neural overlap between reinforcement learning and social conformity may be greater in tasks where a correct answer is objectively defined.^10^ As a result, the consequences of loneliness‐related changes in predictive processing may vary depending on the type of task. For example, in tasks requiring subjective judgments, lonely individuals may experience heightened prediction errors due to uncertainty, leading to a higher likelihood of conforming to others’ answers. On a neural level, this could result in increased activity in areas such as the ventral striatum and insula, which are involved in prediction error monitoring and emotion processing. In contrast, tasks with objective answers (e.g., line judgment task) that reduce prediction errors may lead to less conformity. However, given that chronic loneliness is linked to hippocampal atrophy and memory decline,^126^ chronic loneliness may lead to less accurate predictions and more prediction errors on memory‐related tasks. Consequently, social conformity might be more pronounced in memory‐related tasks in the chronically lonely.

### Context, sex, and attachment in conformity and loneliness

The likelihood of conformity can vary based on whether the influence occurs in a private or public setting and whether it comes from a minority or majority group.^116^ In public settings, where social scrutiny is possible, prediction errors related to social judgment might be amplified, increasing the likelihood of conformity. Influence from a larger group may result in stronger prediction errors between lonely individuals’ own skewed beliefs and the majority opinion. This discrepancy may result in greater social conformity as individuals seek to reduce prediction errors. On a neural level, this may involve increased activation in areas related to error monitoring, social cognition, and the perception of social norms, such as the mPFC and DMN.

Additionally, although not directly studied in the context of loneliness, stress levels may modulate a person's propensity to socially conform by increasing cognitive load and emotional arousal, thereby amplifying prediction errors in social contexts.^127^

We propose that there may be sex‐specific differences in both loneliness and social conformity,^118^ potentially due to variations in socialization and hormonal influences.^71^ For example, a meta‐analysis revealed that women valued interpersonal relationships and harmony more than men. This difference was smaller in collectivist cultures (valuing group harmony) than in individualistic ones (prioritizing individual goals), suggesting cultural influence.^128^

Finally, attachment style may play a mediating role as well. Research suggests that lonely individuals often exhibit insecure attachment styles^88^ characterized by anxious or avoidant behaviors and hindering secure social bonds.^129^ Insecure attachment styles reduce self‐confidence and difficulties in navigating social interactions,^130^ potentially exacerbating social withdrawal and reducing conformity.

### Implications of this model

While this perspective may apply broadly, it requires empirical testing to validate its nuances. For instance, future studies should investigate how loneliness‐related cognitive biases, like maladaptive social priors, affect social interactions and possibly involve the mPFC and insula. This approach could also guide the development of targeted interventions, such as programs aimed at adjusting these biases, and ultimately offering more effective ways to reduce loneliness. By understanding and leveraging the involvement of specific brain regions or networks, we can investigate the negative impacts of loneliness on the brain, identify adaptive strategies, understand neural substrates of decisions with prosocial and harmful outcomes, and develop insights into effective communication and intervention strategies.

Additionally, understanding loneliness and its links to conformity or nonconformity might clarify societal phenomena, such as tendencies toward certain political opinions and directions. Examining how loneliness and social conformity interact within a predictive processing framework provides insights into how social and cognitive biases shape political attitudes and behaviors. Studies show that social exclusion and loneliness increase susceptibility to radicalization and extremism.^131^ Recognizing these influences can inform interventions aimed at addressing loneliness and its effects, ultimately promoting social cohesion and resilience. Such efforts can foster inclusivity and harmony within communities.

Importantly, we do not claim that the proposed model covers all relevant mediating factors as further relevant mechanisms may become evident in empirical studies. It is equally crucial to acknowledge that this model may be sensitive to various moderators, such as group size, the nature of the tasks individuals are engaged in, and the context in which actions are performed (e.g., public vs. private settings). The processes described in our model and illustrated in Figures 1 and 2 are conceptualized as continuous and interdependent, where internal models, external social cues, and behaviors like social conformity dynamically interact over time. Rather than being strictly causal or independent, these processes may form a feedback loop. We propose that loneliness involves reduced precision in forming priors and detecting prediction errors, decreasing model update likelihood.

## INTERVENTIONS FOR COGNITIVE AND NEURAL MECHANISMS IN LONELINESS

Targeted interventions could help address these challenges by introducing cognitive strategies to enhance flexibility, such as cognitive reappraisal, which may boost positive mood during isolation.^132^ Importantly, chronicity of loneliness likely influences treatment outcomes. Testing interventions at different loneliness stages could identify optimal timing and prevent progression to chronic loneliness.

In conclusion, the suggested predictive model of loneliness aims to provide a basic structure and understanding of how acutely to chronically lonely people may demonstrate aberrant predictive processes involving top‐down perceptual discrepancies emanating from areas such as the mPFC and bottom‐up precision of information involving areas such as the insula. While short‐term loneliness may increase the susceptibility to social conformity, persistent loneliness may result in nonconformity. Empirical research is essential to test this model and explore the heterogeneity of loneliness, as well as to further demonstrate which factors and contexts may lead to social conformity or nonconformity in lonely individuals.

## AUTHOR CONTRIBUTIONS

D.S. and N.H. conceived the idea for the perspective. D.S., N.H., and D.‐M.L. planned the paper, outlined the key components of the perspective, and drafted the proposed model. D.‐M.L. refined the figures. H.B. advised on theoretical perspectives. N.H. wrote the draft of the original manuscript. All authors contributed to revising and editing the manuscript.

## COMPETING INTERESTS

The authors declare no competing interests.

### PEER REVIEW

The peer review history for this article is available at https://publons.com/publon/10.1111/nyas.15324.
